# Synergistic effect of co-exposure to carbon black and Fe_2_O_3 _nanoparticles on oxidative stress in cultured lung epithelial cells

**DOI:** 10.1186/1743-8977-6-4

**Published:** 2009-02-09

**Authors:** Bing Guo, Rema Zebda, Stephen J Drake, Christie M Sayes

**Affiliations:** 1 Department of Mechanical Engineering, Texas A&M University, College Station, Texas 77843, USA; 2 Department of Veterinary Physiology & Pharmacology, Texas A&M University, College Station, Texas 77843, USA

## Abstract

**Background:**

There is a need to better understand synergism in the biological effects of particles composed of multiple substances. The objective of this study was to determine if the oxidative stress in cultured cells caused by co-exposure to carbon black and Fe_2_O_3 _nanoparticles was significantly greater than the additive effects of exposure to either type of particles alone; and to determine a possible cause for such synergistic effect if one was found. Cultured A549 human lung epithelial cells were exposed to (1) carbon black nanoparticles alone, (2) Fe_2_O_3 _nanoparticles alone, and (3) both types of particles simultaneously. Protein oxidation, lipid peroxidation, and cellular uptake of Fe in these cells were measured after 25 hours of exposure. The reduction of solubilized Fe^3+ ^by the carbon black nanoparticles was measured separately in a cell-free assay, by incubating the carbon black and the Fe_2_O_3 _nanoparticles in 0.75 M sulfuric acid at 40°C and measuring the amount of reduced Fe^3+ ^at different time points up to 24 hours.

**Results:**

Cells exposed to carbon black particles alone did not show protein oxidation, nor did the cells exposed to Fe_2_O_3 _particles alone, relative to the control. However, cells co-exposed to both carbon black and Fe_2_O_3 _particles showed up to a two-fold increase in protein oxidation relative to the control. In addition, co-exposure induced significant lipid peroxidation, although exposure to either particle type alone did not. No significant difference in cellular iron uptake was found between single exposure and co-exposure, when the Fe_2_O_3 _dosing concentration was the same in each case. In the cell-free assay, significant reduction of Fe^3+ ^ions by carbon black nanoparticle was found within 2 hour, and it progressed up to 24 hours. At 24 hours, the carbon black nanoparticles showed a reductive capacity of 0.009 g/g, defined as the mass ratio of reduced Fe^3+ ^to carbon black.

**Conclusion:**

Co-exposure to carbon black and Fe_2_O_3 _particles causes a synergistic oxidative effect that is significantly greater than the additive effects of exposures to either particle type alone. The intracellular redox reaction between carbon black and Fe^3+ ^is likely responsible for the synergistic oxidative effect. Therefore elemental carbon particles and fibres should be considered as potential reducing agents rather than inert materials in toxicology studies. Acidified cell organelles such as the lysosomes probably play a critical role in the solubilization of Fe_2_O_3_. Further research is necessary to better understand the mechanisms.

## Background

An increasing body of scientific research has led researchers to the conclusion that exposure to fine particulate air pollution has adverse effects on human health. [1] This conclusion appears to be soundly based on statistical studies that have reported correlation between airborne particulate matter (PM) concentration and adverse health effects in humans [2,3]. However, important gaps still remain in the scientific understanding about detailed mechanism of PM toxicity. Since the early 1990s when epidemiological results became accepted, research has produced many possible mechanisms for the toxicity of particulate matter. Still, no known chemical component of the particulate matter is of sufficient toxicity at the given exposure level to explain the observed health effects. [4] The toxicity of PM based on data from the epidemiological studies appears to be much greater than the toxicity estimated by adding the known toxicity values of the individual substances in a "typical" PM composition. There are a number of possible explanations for this discrepancy. One of them is synergism between the different components of particulate matter. [5] Synergism is well known in medicine, a common example being that between alcohol and other drugs. Synergistic effects of co-exposure to asbestos and other chemical substances have also been reported. [5] Although synergism may not answer all the questions regarding particle and fibre toxicity, it is evidently an important factor that deserves better understanding given that exposure almost always involves not one, but multiple chemical substance.

In this study, we used carbon black nanoparticles and Fe_2_O_3 _nanoparticles as model materials to investigate the synergistic biological effect. The two particle types were selected because of their relevance to airborne particulate matter, and the possibility of synergism between them. Carbon black particles have been used extensively in toxicology studies as a surrogate for elemental carbon in airborne particulate matter; while Fe_2_O_3 _nanoparticles have been used to represent transition metals in particulate matter. Elemental carbon and transition metals are commonly found in urban atmospheric aerosols. For example, a study in seven southern California cities found that atmospheric ultrafine (< 100 nm) particles were composed of (by mass) 8.7% elemental carbon and 14% transition metal oxides, with the rest being mostly organic compounds and sulphate and nitrate [6]. Toxicology experiments have suggested synergism may exist between carbon black particles and transition metals. Wilson *et al*. [7] measured the generation of reactive oxygen species (ROS) in cell-free systems and cell culture by soluble transition metals and ultrafine (14 nm) carbon black particles. They found significant synergistic effect between the transition metals and the ultrafine carbon black particles. However, so far no explanation has been offered for the observed synergistic effect between carbon black and transition metals. Also, there has not been sufficient research on the synergistic effect between carbon black particles and water-insoluble metal-containing particles, such as metal oxides particles.

The toxicity of certain particles, such as those derived from oil fly ash, has been associated with the water-soluble metals in the particles. [8] However, water-insoluble metals may also play an important role in the biological effects of airborne particulate matter. Some studies have found that water-insoluble metals, not water-soluble metals, are responsible for adverse biological effects. For example, Metzger *et al*. [9] found no correlation between cardiovascular emergency department visits and water-soluble metals. (Insoluble metal data was not available from that study.) Prahalad *et al*. [10] exposed human monocytes and polymorphonuclear leukocytes to ambient aerosol particles. They found no correlation between reactive oxygen species (ROS) generation and water-soluble metals, but significant correlation between ROS and water-insoluble transition metals including Fe. Costa and Dreher found that inflammatory response in rats were largely in proportion to the amount of acid-soluble metals in different types of PM samples [11]. Iron is one of the most abundant transition metals in airborne particles [12]. Fe_2_O_3 _particles are insoluble in water, but soluble in acids. [13] Therefore they would be good representatives of the airborne particles that contain water-insoluble transition metals.

We hypothesized that synergism between carbon black and Fe_2_O_3 _nanoparticles might take effect through the oxidative stress paradigm. Oxidative stress is the dominant model used for particle toxicology[14], and has been primarily attributed to the bioavailable transition metals [15]. For example, hydroxyl radicals may be generated through reactions that involve transition metal ions, such as the Fe^2+ ^ion, through the Fenton reaction:

Fe^2+ ^+ H_2_O_2 _→ Fe^3+ ^+ OH· + OH^-^

The hydroxyl radical OH· is highly reactive and can react with biological molecules to cause oxidative stress. This reaction is highly dependent of the availability of Fe^2+ ^ions. Redox "cycling" has been invoked as a mechanism for "sustained" oxidative stress, in which an electron donor reduces Fe^3+ ^ions to Fe^2+ ^ions, so that the Fenton reaction shown above can continue for a prolonged period of time [16]. Biological species such as ascorbate or environmental pollutants such as quinoid species have been considered as being able to cause such redox "cycling". Carbon black particles may also cause redox "cycling" by reducing Fe^3+ ^to Fe^2+^. Activated carbons [17,18] and carbon nanotubes [19] have been shown to reduce metal ions, including Fe^3+ ^(reduced to Fe^2+^). This reduction capability has been attributed to reactive functional groups on the carbon surface. For example, the Fe^3+ ^may be reduced to Fe^2+ ^by elemental carbon through the following reaction [20]:

~C-OH + Fe^3+ ^→ ~C = O + Fe^2+ ^+ H^+^

In this reaction, a hydroxyl group ~C-OH on the carbon surface is oxidized and becomes a carboxyl group ~C = O. In the mean time, a Fe^3+ ^ion is reduced to become a Fe^2+ ^ion, accompanied by the generation of a proton.

It is intuitive to predict that the carbon black particles commonly used for toxicity studies can also reduce Fe^3+ ^in aqueous solutions. However, such reaction has not been reported until now. More significantly, until this work the role of the carbon-black/Fe^3+ ^redox reaction in particle biological effects had never been investigated.

**Figure 1:**
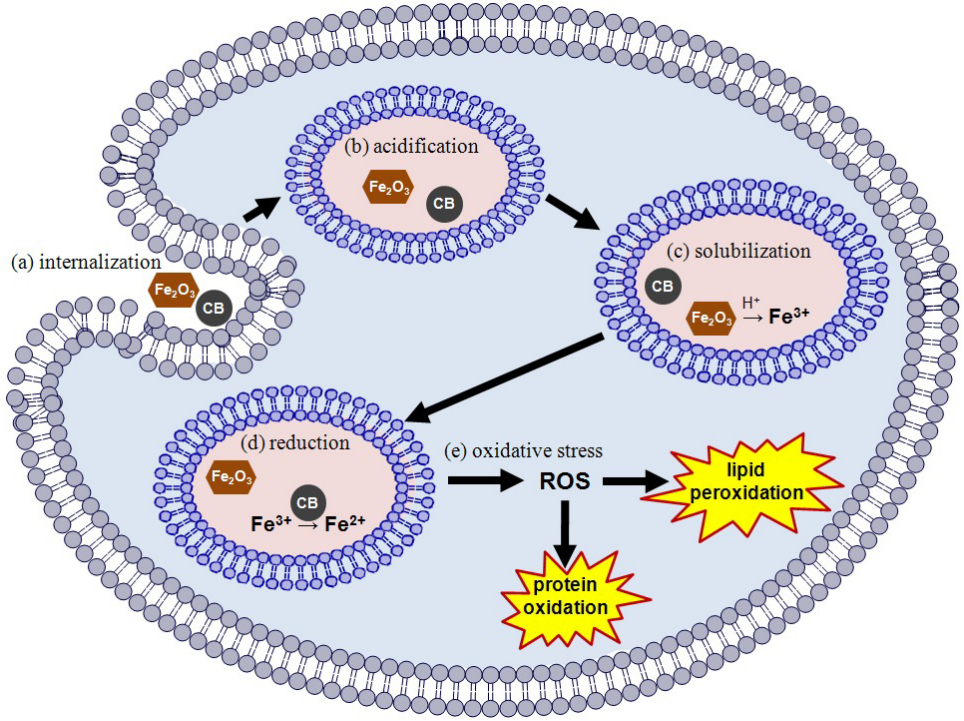
**Illustration of events following concurrent endocytosis of both carbon black nanoparticles and Fe_2_O_3 _nanoparticles**.

We hypothesized that the redox reaction between carbon black particles and bioavailable Fe^3+ ^could take place within a cell, and the reduction of bioavailable Fe^3+ ^would result in a synergistic effect. As a result, co-exposure to carbon black and Fe_2_O_3 _nanoparticles would cause significantly greater oxidative stress than the additive effects due to exposure to either particle type alone. This hypothesis was based on the assumptions that (1) in single exposure to Fe_2_O_3 _particles, the iron would become bioavailable in the trivalent state, and hence would not generate hydroxyl radicals through the Fenton reaction, and (2) in single exposure to carbon black nanoparticles, the reductive capacity of carbon black would be restrained due to the nature of intracellular uptake of particles. For the co-exposure to both carbon black and Fe_2_O_3 _nanoparticles, a mechanistic model might be proposed to account for the synergistic effect. This is illustrated in Figure 1 and described in detail in the Discussion section. Herein we report the experimental results, interpretation of the results, conclusions, and the experimental methods.

## Results

### Particle Size, Surface Area and Morphology

**Figure 2:**
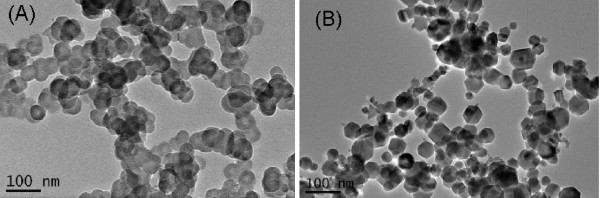
**TEM images of (A) the carbon black nanoparticles and (B) the Fe_2_O_3 _nanoparticles**.

Fe_2_O_3 _nanoparticles and carbon black nanoparticles were synthesized by flame methods as described in the Methods section. The TEM images of the carbon black particles and the Fe_2_O_3 _particles are shown in Figure 2. The carbon black particles are aggregates that consisted of spherical primary particles. The Fe_2_O_3 _particles were faceted and non-agglomerated, with a size distribution similar to what had been found in a previous work [21]. The specific surface areas for the carbon black particles and the Fe_2_O_3 _particles are 63 m^2^/g and 49 m^2^/g, respectively. The calculated surface area mean diameter (the Sauter diameter) for the carbon black particles and the Fe_2_O_3 _particles was 52.8 nm and 23.5 nm, respectively. A survey of the TEM images showed that the mean diameter of the carbon black primary particles was 47 nm, with standard deviation 7 nm. The mean particle diameter thus obtained for the Fe_2_O_3 _particles was 41 nm, with standard deviation 17 nm. It should be noted that particles smaller than 10 nm were present in the Fe_2_O_3 _particle sample, which could not be adequately quantified by surveying the TEM image. If those very small (<10 nm) Fe_2_O_3 _particles were fully accounted for, the mean diameter for the Fe_2_O_3 _particles should be in closer agreement with the Sauter diameter calculated from the BET result. This phenomenon has been described in detail elsewhere [21].

### Protein Oxidation for Single Exposures and Co-exposures

**Figure 3:**
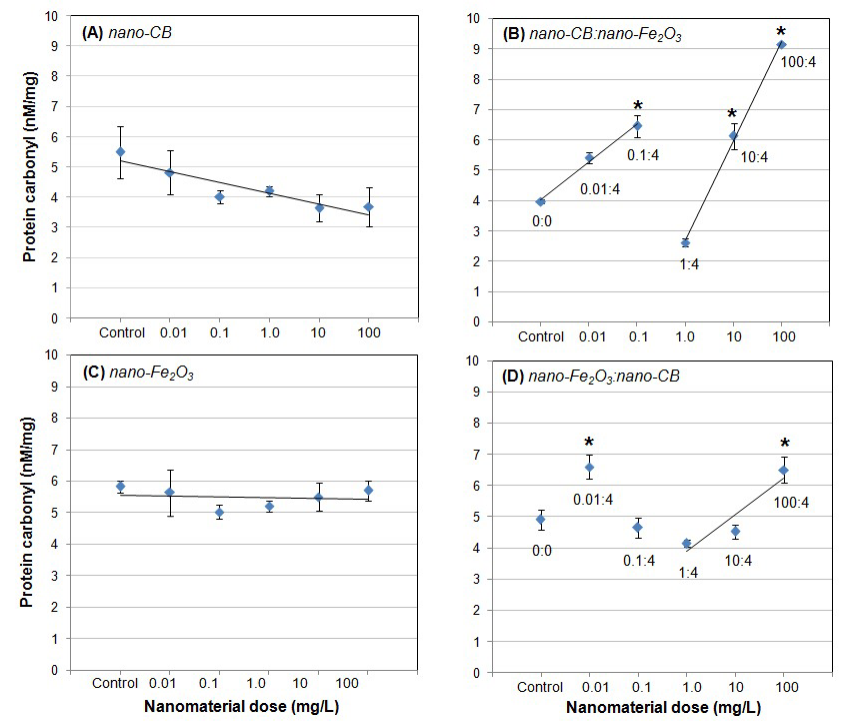
**Protein carbonyl concentrations in A549 cells after 25 hours of exposure to (A) carbon black nanoparticles alone, (B) carbon black nanoparticles and Fe_2_O_3 _nanoparticles, (C) Fe_2_O_3 _nanoparticles alone, and (D) Fe_2_O_3 _nanoparticles and carbon black nanoparticles**. Numbers within the co-exposure graphs denote doses of the two materials. "Nanomaterial dose" refers to the concentration of the nanoparticles in the cell culture medium. Values given are means ± SD (*p < 0.05 relative to control cell population).

Human lung epithelial cells exposed to either carbon black alone or Fe_2_O_3 _alone did not show statistically significant protein oxidation increase relative to the control, as seen in Figure 3(A) and 3(C). Protein oxidation in cells treated with carbon black even showed a decreasing trend with increasing dose of carbon black in comparison to the control. However, cells co-exposed to carbon black and Fe_2_O_3 _showed significant protein oxidation, as seen in Figure 3. As shown in Figure 3(B), the protein oxidation increase for carbon black concentrations 0.1, 10, and 100 mg/L (Fe_2_O_3 _4 mg/L) was statistically significant (p < 0.05). The increase in protein oxidation at Fe_2_O_3 _concentrations 0.01 and 100 mg/L (carbon black 4 mg/L) was also statistically significant, as shown in Figure 3(D),

In single exposures, the protein oxidation was always at the control level (or even lower) regardless of the single exposure particle concentration. Consequently, the additive effect of single exposures was at most zero relative to the control. Therefore, in the co-exposure cases, the protein oxidation also would be significantly greater than the additive effect of the corresponding single exposures.

### Lipid Peroxidation Results

**Figure 4:**
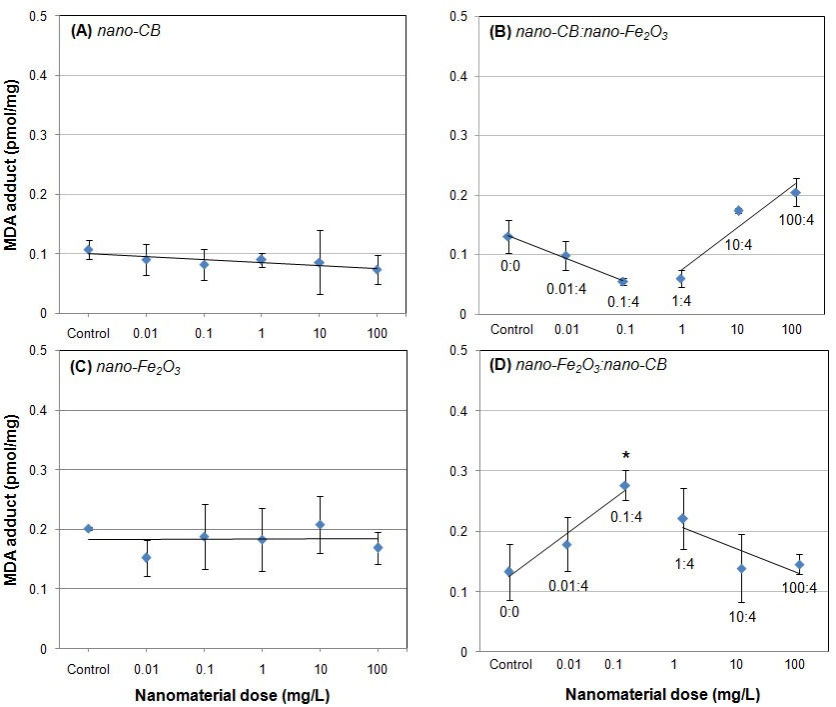
**Malondialdehyde-lipid adduct (MDA-adduct) levels in A549 cells after 25 hours of exposure to (A) carbon black nanoparticles alone, (B) carbon black nanoparticles and Fe_2_O_3 _nanoparticles, (C) Fe_2_O_3 _nanoparticles alone, and (D) Fe_2_O_3 _nanoparticles and carbon black nanoparticles**. Numbers within the co-exposure graphs denote doses of the two materials. "Nanomaterial dose" refers to the concentration of the nanoparticles in the cell culture medium. Values given are means ± SD (*p < 0.05 relative to control cell population).

Exposure to either particle type alone did not induce statistically significant lipid peroxidation, as seen in Figure 4. However, cells co-exposed to carbon black and Fe_2_O_3 _showed significant protein oxidation. In Figure 4(D), the lipid peroxidation at Fe_2_O_3 _concentrations 0.1 mg/L (carbon black 4 mg/L) was significantly higher than that of the control. The lipid peroxidation for this co-exposure case also would be significantly greater than the additive effect of the corresponding single exposures. The lipid peroxidation values for other co-exposure cases were not statistically significant than the control. However, up and down trends were observed in the co-exposures, which were clearly different than the single exposures.

### Reduction of Dissolved Fe^3+ ^by Carbon Black Nanoparticles

**Figure 5:**
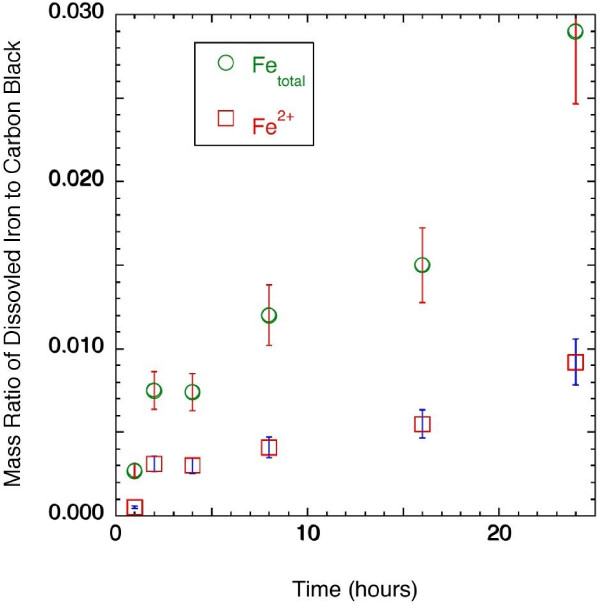
**The mass of dissolved Fe^2+ ^and Fe^3+ ^ions normalized to carbon black mass as a function of time when the carbon-black/Fe_2_O_3 _particles was incubated in 0.75 M H_2_SO_4 _(100 mL) at 40°C for different times**.

The reduction of Fe^3+ ^by carbon black nanoparticles was measured in a cell-free assay. When carbon black and Fe_2_O_3 _particles were co-incubated in 0.75 M H_2_SO_4_, both Fe^3+ ^and Fe^2+ ^ions were found in the solution. The mass of dissolved Fe^2+ ^and Fe^3+ ^per unit mass of carbon black are shown in Figure 5 as functions of incubation time. The results showed that the total iron (Fe^3+ ^and Fe^2+^) and Fe^2+ ^concentrations in the acidic solution both continued increasing throughout the 24 hours. When Fe_2_O_3 _particles alone were incubated in 0.75 M H_2_SO_4 _for 8 hours, only Fe^3+ ^ions were found. This showed that, when carbon black particles were present, the Fe^3+ ^ions derived from solubilization of Fe_2_O_3 _were reduced to Fe^2+ ^ions.

No dissolved iron was found when carbon black alone was incubated in 0.75 M H_2_SO_4 _for 8 hours. This suggests that the acid-leachable iron impurity in the carbon black particles, if any, was below the detection limit of the method (0.05% Fe/carbon mass ratio, assuming a lowest discernable absorbance reading of 0.002). Neither was dissolved iron detected in the resulting solution from the method blank (acid solution incubated in glass beaker with no particles added). This showed that the acid-leachable iron from the beakers was below detection limit of the method. In addition, a mass balance calculation showed that over 90% of the iron from the original Fe_2_O_3 _particles was recovered from the solution after the acid incubation. This suggested that the losses in this process, including absorption of iron by the filter, were reasonably low.

### Cellular Iron Uptake Results

The quantitative cellular iron uptake was obtained indirectly by comparing the iron content in the cell culture medium before and after cell exposure (Table 1). The total iron contents in the Fe_2_O_3_-spiked medium before exposure and in the "spent" medium after single exposure and co-exposure were measured by ICP-AES. The inherent iron content in the pristine medium was also measured. The results are given in Table 1. The single exposure spent medium was derived from 7 mL of medium that had been spiked with 3.5 μg of Fe_2_O_3 _(2.45 μg Fe), in which cells were incubated for 25 hours. The co-exposure spent medium was derived from 7 mL of medium that had been spiked with 3.5 μg of Fe_2_O_3 _and 3.5 μg of carbon black, in which cells were incubated for 25 hours. The iron content in the 7 mL cell culture medium spiked with 3.5 μg Fe_2_O_3 _(before cell exposure) was measured separately, so was the inherent iron content in 7 mL pristine cell culture medium. By subtracting the iron content in the spent medium from that in the Fe_2_O_3_-spiked medium, we could estimate the cellular iron uptake. Thus estimated iron uptake was 2.52 ± 0.14 μg for the single exposure and 2.66 ± 0.14 μg for the co-exposure. The results suggested that there was no statistically significant difference in the cellular iron uptake between single exposure and co-exposure, when the iron dosing concentration was kept the same in each case.

**Table 1:** Total iron contents in cell culture medium samples

Samples	Single exposure spent medium	Co-exposure spent medium	Medium spiked with Fe_2_O_3_	Pristine medium
Total iron content in medium (μg) *	0.84 ± 0.07	0.70 ± 0.07	3.36 ± 0.07	1.26 ± 0.07
Inferred cellular iron uptake (μg) **	2.52 ± 0.14	2.66 ± 0.14	NA	NA

* The uncertainty value only reflects the instrument uncertainty.

** Obtained by subtracting the iron in the spent medium from that in the medium spiked with Fe_2_O_3_.

## Discussion

### Synergistic Oxidative Stress due to Co-exposure

The protein oxidation and the lipid peroxidation results clearly showed that co-exposure had a synergistic effect on oxidative stress. The non-monotonous relation between the oxidative endpoints and the dosage of exposure suggested that complex cellular response, such as antioxidant enzyme production, might have been triggered at certain nanomaterial concentration and/or certain time of co-exposure, and apparently such cellular anti-oxidation response was then overwhelmed at excessively high nanomaterial concentrations and/or prolonged continual co-exposure. Nevertheless, the oxidative stress in co-exposures was significantly greater than the sum of protein oxidation due to exposures to the individual particle types at the corresponding concentrations. Previously in an *in vivo *study, Zhou *et al. *had investigated the effect of co-exposure to iron oxide particles and carbon black (flame soot) in comparison to exposures to either particle type alone [22]. They also found some synergistic oxidative effect due to co-exposure. The difference between this study and the work by Zhou *et al*. is: in this work, the same carbon black particles (or Fe_2_O_3 _particles) were used in the single exposures and the co-exposures; where in the work by Zhou *et al*. the particles used in the single exposures were synthesized under different conditions than those used in the co-exposures. In fact the authors suggested that the iron oxide particles used for the co-exposures might have different oxidation state than that for the single exposure [22]. In this study, we were able to specifically focus on the synergistic biological effect of the two types of particles.

In this study the carbon black particles alone did not cause increase in protein oxidation. This was in agreement with many previous studies. For example, Gurgueira *et al*. [23] measured lung tissue spontaneous chemiluminescence as an indicator of oxidative stress caused by concentrated airborne particles and carbon black (no detectable transition metals) in rats through inhalation exposure, and the carbon black showed no significant oxidative stress. Some researchers such as Lam *et al*. [24] used carbon black (no detectable metals) as negative control in animal inhalation exposure studies. Xia *et al*. found no H_2_O_2 _generation (based on a nanobiosensor) in cultured murine macrophages treated with carbon black (no detectable metal) [25].

However, in other studies, carbon black particles had been found to cause oxidative stress. Stone *et al*. found significant generation of reactive oxygen species production using supercoiled plasmid DNA in human alveolar epithelial cells due to carbon black nanoparticles (metal concentration said to be low but not specified) [26]. Pulskamp *et al*. found reactive oxygen species generation in cells treated with carbon black (metal concentration unspecified) [27]. Generally, in the studies where carbon black was found to cause no oxidative stress, metal impurities were shown to be below detection limit; while in those where carbon black was found to cause oxidative stress, metal impurity information was generally not available. Therefore it is possible that the results were confounded by metal impurities, when carbon black was found to cause oxidative stress. The discrepancy in the literature suggests the need to determine possible confounding factors, such as the synergistic effect between carbon black and transition metals.

The Fe_2_O_3 _particles did not cause protein oxidation in this study. This result was in line with a number of other studies. For example, Gojova *et al. *found that Fe_2_O_3 _nanoparticles did not cause inflammatory response in cultured human vascular endothelial cells [28]. Zhou *et al. *[22] did not find significant oxidative stress in rats exposed to Fe_2_O_3 _nanoparticles through inhalation. This might be explained by that the bioavailable iron from Fe_2_O_3 _particles was Fe^3+^, which was inactive in generating reactive oxygen species, and hence did not cause oxidative stress.

### Reduction of Fe^3+ ^by Carbon Black Particles

To propose a mechanism for the synergistic biological effect of co-exposure, one would need to identify the possible interactions between carbon black particles and Fe_2_O_3 _particles. Our results identified one of such interactions, that is, the carbon black nanoparticles were able to reduce solubilized Fe^3+ ^(from Fe_2_O_3_) into Fe^2+^. In fact, one could calculate the reductive capacity of the carbon black nanoparticles based on the amount of the resultant Fe^2+^. The results are shown in Table 2. As can be seen in Table 2, the reductive capacity of the carbon black nanoparticles was similar to the reductive capacity of activated carbon obtained in a previous study [17].

**Table 2:** Mass-based and surface area-based reductive capacity of the carbon samples

	Reduced Fe^3+ ^per unit mass carbon (g/g)	Electrons donated per unit mass carbon (mEq/g carbon)
This work (at 25 h)	0.009	0.16
Shirakashi [17] (at 20 h)	0.008	0.15

Since the reductive capacity of the elemental particles was related to the surface functional groups, they would lose their reductive capacity once the reductive surface functional groups were all used. Therefore, pre-oxidized carbon black particles would have lower reductive capacity than as-synthesized carbon black particles.

### Iron Solubilization and Possible Role of Acidified Intracellular Vesicles

Fe_2_O_3 _particle solubilization would be necessary for Fe to be available to the cells, and for any potential Fe^3+ ^reduction by carbon black to occur. At pH levels of cell growth medium, the solubility of ferric iron is very low unless it is bound with chelators. Therefore Fe_2_O_3 _particles were unlikely to solubilized in cell growth medium. (Fe_2_O_3 _is insoluble in water.) However, Fe_2_O_3 _particles could become solubilized within a cell. Once particles were internalized, they would be transported through endosomes and eventually delivered to lysosomes for final degradation [29]. Endosomes and lysosomes are found in various cell types including epithelial cells [30,31] and endothelial cells [32]. The interior of endosomes and lysosomes is acidic, with pH as low as 4.5 [32,33]. Fe_2_O_3 _particles, although insoluble in water, would be solubilized in the acidic cell organelles. Iron thus would become available in the form of Fe^3+ ^ions if there was no reductant present. When a mixture of Fe_2_O_3 _particles and carbon black particles were internalized simultaneously, then the carbon black nanoparticle might reduce the bioavailable Fe^3+ ^ions within a living cell. A simplified illustration of this iron availability mechanism is shown in Figure 1.

Positive identification of the particle-containing vesicles and their detailed functions was beyond the scope of this study. Therefore, we did not make any attempt to manipulate lysosomal functions in order to test our hypothesis. Whereas, it is our opinion that the role of acidified vesicles such as lysosomes in the solubilization of water insoluble particles has not been properly recognized in the particle and fibre toxicology literature, and it should be further investigated.

### Proposed Mechanism for Synergism

Here we give an explanation for the synergistic effect of co-exposure on oxidative stress. Referring to Figure 1, when cells were exposed to Fe_2_O_3 _particles alone, the Fe_2_O_3 _particles were internalized intracellularly and they became solubilized in acidified vesicles, such as lysosomes, to produce Fe^3+ ^ions. The Fe^3+ ^ions would not induce significant oxidative stress, because Fe^3+ ^ions would not generate hydroxyl radicals in the presence of H_2_O_2 _unless a reductant was present. On the other hand, when cells were exposed to carbon black particles, the carbon black particles were internalized intracellularly and contained in the acidified vesicles. The reductive capacity of the carbon black particles was restricted because they were bound within the vesicles. However, when cells were co-exposed to carbon black and Fe_2_O_3 _particles simultaneously, a synergistic effect might take place, as partly illustrated in Figure 1. Carbon black and Fe_2_O_3 _particles would be internalized together, and stored in the acidified vesicles together. The Fe_2_O_3 _would be solubilized to produce Fe^3+ ^ions. Some of the Fe^3+ ^ions would then be reduced by the carbon black particles and became Fe^2+ ^ions. When the Fe^2+ ^ions permeated the vesicle membrane, they could take part in the Fenton reaction and then induce protein oxidation. Thus, co-exposure to carbon black and Fe_2_O_3 _particles would cause oxidative stress that was significantly greater than the additive effect of exposures to either carbon black or Fe_2_O_3 _alone.

Although in this work we used carbon black particles and Fe_2_O_3 _particles, it is worth noting that similar synergistic effect should be expected for other types of elemental carbon particles (e.g. activated carbon, C-60, carbon nanotubes, etc.) and other transition metal oxide particles. It should be noted that other researchers have related the redox activities of ambient particles (containing various substances) to their biological effects [34]. Also, the redox activities of ambient aerosol particles have been associated with the elemental carbon content in the particles [35]. However, to the best of our knowledge, in particle and fibre toxicology this study is the first to specifically identify a mechanism for the synergism between elemental carbon particles and transition metal oxide particles, to explicitly recognize elemental carbon as a possible reducing agent rather than an inert material, and to hypothesize the role of acidified cell organelles in solubilizing water-insoluble metal oxide particles and hence the availability of metals.

As discussed previously, the reductive capacity of elemental carbon materials is related to the surface functional groups. Therefore, surface modifications of the elemental carbon materials may change the reductive capacity and hence alter the synergistic oxidative effect between elemental carbon and transition metals.

### Other Factors

#### 1. Cell culture medium

The cell culture medium contained iron and copper. Since the hypothesized mechanism involved bioavailable iron, the potential effect of the cell culture medium should be addressed. Here we focus only on the possible interaction between carbon black and iron in the medium. Further we assume that the iron from the fetal bovine serum was completed bound with chelators and non-reactive towards carbon black. Then the only reactive iron was that in the F-12K medium. The iron concentration of the F-12K medium was 0.17 mg/L, according to the product datasheet. The source of iron in the F-12K medium was FeSO_4_•7H_2_O. If the iron remained as Fe^2+^, then it would not react with carbon black and hence the reducing capability of the latter should be retained. Fe^2+ ^ions in the presence of air could be gradually oxidized to Fe^3+^. However, the solubility of Fe^3+ ^at physiologically relevant pH levels would be very low, unless it was bound with chelators. The reactivity between chelated Fe^3+ ^and carbon black was unknown. The reaction would be presumably much slower than free Fe^3+ ^ions. Based on the above reasoning, the transition metals in the cell growth medium should not confound the interpretation of results.

#### 2. Naturally occurring Fe and reducing agents in lysosomes

Under "natural" conditions lysosomes contain Fe^3+ ^and Fe^2+ ^and reducing equivalents, and Fenton reaction takes place within lysosomes. [36] Therefore, a significant increase in oxidative stress might be associated with abnormally high Fenton chemistry activities in the lysosomes. Evidently co-exposure could result in such an outcome: the internalized Fe_2_O_3 _particles and their solubilization would elevate the bioavailable iron concentration within the lysosomes; the internalized carbon black (CB) particles would serve as extra reducing equivalents in addition to what naturally existed in the lysosomes. Therefore, although synergism of Fe_2_O_3 _and carbon black might not be needed for naturally occurring Fenton reaction within the lysosomes, this synergism would nevertheless elevate the level of this reaction to an abnormal level. Therefore, the proposed mechanism for the CB/Fe_2_O_3 _synergism is consistent with the current understanding about lysosomes.

#### 3. Effect of co-exposure on particle uptake

Co-exposure might have some effect on cellular uptake. For instance, the cellular uptake of Fe_2_O_3 _nanoparticles might be dependent on whether it was single exposure or co-exposure. Therefore, the synergistic effect could potentially be attributed to enhanced particle uptake that was associated with co-exposure. We did not have the means to quantify the uptake of carbon black. Nevertheless, the iron uptake results suggested that there was no significant difference in iron uptake between single exposure and co-exposure, when the Fe_2_O_3 _dose was kept constant. Therefore, the effect of co-exposure on particle uptake, if any, was not sufficient to explain the observed synergistic oxidative stress.

#### 4. Cellular response to co-exposure induced oxidative stress

In this study, non-monotonous relationship was found between the biological response and the co-exposure dosage. In other words, the synergistic effect of co-exposure did not always increase with increasing dosage. Such non-monotonous response-dosage relationship could also be found from among the limited number of similar studies. In the work by Wilson *et al*., cell culture was co-exposed to carbon black particles and ZnCl_2_, and was exposed to ZnCl_2 _alone. At lower ZnCl_2 _concentrations, the co-exposure induced significant TNF-α generation, compared with exposure to ZnCl_2 _alone. However, at higher ZnCl_2 _concentrations, the difference between co-exposure and exposure to ZnCl_2 _alone was not significant. [7]

Non-concordance existed between the protein oxidation and the lipid peroxidation results in this study. That is, at a particular exposure condition, significant response might be found for one biological endpoint but not for the other. Similar non-concordance could be found from many particle toxicology studies. For example, in the work by Wilson *et al*., significant TNF-α production was found for co-exposure to carbon black and ZnCl_2_, compared with exposure to ZnCl_2 _alone, but similar co-exposure did not induce significantly greater oxidation of DCFH to DCF than exposure to ZnCl_2 _alone. [7] In the work by Zhou *et al*., the co-exposure to iron oxide particles and carbon black particles induced significant response in the GSSG level, but not in the GSH level. [22]

As our results suggested, the cellular response to co-exposure, as measured with the oxidative stress endpoints, was a complex one. This should not be surprising because the cell response involves mechanisms yet to be explored. It might be reasonable to conjecture that factors such as dosage, composition, and time all played a role in the measured responses. The functional relationship between the response and the factors was not a monotonous one. A combination of conditions might exist that would have minimized biological impact. Understanding of such functional relationship is probably very useful for mitigating the adverse health effect of nanomaterials and should be further investigated.

## Conclusion

Co-exposure to carbon black and Fe_2_O_3 _particles can cause oxidative stress that is significantly greater than the additive effects of exposures to either particle type alone. This synergistic effect is very likely due to the reduction of Fe^3+ ^ions by carbon black nanoparticles within the cell. Further, the redox activities between elemental carbon particles and bioavailable metal ions may play a significant role in the toxicity of ambient airborne particulate matter. In particle and fibre toxicology studies, elemental carbon materials should be considered as potential reducing agents rather than inert materials. Surface modifications of the elemental carbon particles/fibres may significantly change their reductive capacity, and hence their toxicological behavior. The role of acidified intracellular vesicles such as lysosomes in the solubilization of water insoluble metal oxide particles should be further investigated in the particle and fibre toxicology context.

## Methods

The biological endpoints used in this study were protein oxidation and lipid peroxidation in cultured human lung epithelial cells exposed to particles. The cellular uptake of Fe, when applicable, was measured by inductively coupled plasma emission spectroscopy (ICP-AES). The reduction of dissolved Fe^3+ ^ions by carbon black particles was measured by a spectrophotometric method following incubating the carbon black and Fe_2_O_3 _nanoparticles in an acidic solution. The nanoparticles were characterized as described below.

### Nanoparticles Synthesis and Materials Characterization

Both the Fe_2_O_3 _nanoparticles and the carbon black nanoparticles were synthesized using a custom-built flame apparatus (burner nozzle OD/ID 9.5/7.9 mm), which had been described in detail elsewhere [37]. To synthesize Fe_2_O_3 _nanoparticles, H_2 _gas at 1 standard liter per minute (SLM) passed through an iron pentacarbonyl (Fe(CO)_5_) container kept at 23 ± 1°C to be mixed with the Fe(CO)_5 _vapor. The H_2_/Fe(CO)_5 _gas mixture was burned in O_2 _in the form of a co-flow diffusion flame (H_2 _flow in the center with a parallel O_2 _flow at 5 SLM around the H_2_). Iron oxide nanoparticles were generated in the flame and collected on filters. The particles had the crystal structure of maghemite (γ-Fe_2_O_3_). Similar flame-synthesized Fe_2_O_3 _nanoparticles had previously been used in a toxicology study. [28] The detailed synthesis and characterization of those Fe_2_O_3 _was described elsewhere. [21] Flame carbon black (soot) particles were synthesized in a C_2_H_2_/air diffusion flame by a method similar to that used by Yang *et al*. [38] Briefly, acetylene (C_2_H_2_) gas was burned in air in the form of a sooting co-flow diffusion flame. The carbon black (soot) particles were collected on filters. The acetylene (C_2_H_2_) flow rate was 0.35 standard liters per minute (SLM). The supporting air flow rate was 10 SLM. TEM sample preparation was described elsewhere [21]. The particle samples were analyzed in a JEOL 2010 microscope (JEOL Ltd., Tokyo, Japan) operated at 200 kV. Surface area measurement was carried out for the carbon black particles and the Fe_2_O_3 _particles using a Micromeritics ASAP 2020 (Micromeritics Instrument Corp., Norcross, GA). Particle samples were degassed under vacuum overnight. The following morning, the specific surface area was measured using the N_2 _adsorption (BET) method [39].

### Cell Culture and Protein Oxidation Assay

Human lung epithelial cells (A549, American Type Culture Collection, Manassas, VA) were cultured using F-12 K media supplemented with 10% fetal bovine serum and 1% penicillin, streptomycin, and amphotercin B (Sigma Aldrich, St. Louis, MO). Cells were seeded in 24-well plates. After allowing at least 48 hrs for attachment, cells were exposed to carbon black nanoparticles or Fe_2_O_3 _nanoparticles alone, as well as co-exposed to mixtures of carbon black and Fe_2_O_3 _particles. For single exposures, the cells were incubated with carbon black or Fe_2_O_3 _particles in cell culture medium at concentrations from 0.01 mg/L to 100 mg/L. For co-exposures, the concentration of one type of particle was kept constant at 4 mg/L, while the other one varied from 0.01 mg/L to 100 mg/L. Stock suspension in ultrapure water of each type of particle (10 mg/mL) was sonicated for 45 minutes prior to dilution in cell culture medium [28,40]. A549 cells incubated with particle-free culture medium served as the controls. The level of protein oxidation was determined using the Oxiselect™ Protein Carbonyl Elisa Kit (Cell Biolabs, Inc., San Diego, CA) by measuring the amount of carbonyl derivatives [41]. The protein oxidation assay was applied after 25 hours of exposure. Protein oxidation in A549 cells was measured using the dinitrophenylhydrazine (DNPH) assay probing for protein carbonyl groups, the most common product of protein oxidation [42], and normalized to total protein. Cells were homogenized in 1 × PBS (pH 7.2) then sonicated for 20 minutes for lysis and protein release. Extracted protein was then plated on protein binding plate, anti-DNPH antibody and secondary antibody were added to detect protein carbonyl. Absorbance was read at 450 nm on a Multiskan MCC plate reader (Thermo Electron Corp., Waltham, MA).

### Lipid Peroxidation Assay

Lipid peroxidation was measured in A549 cells using the thiobarbituric acid (TBA) assay for malondialdehyde (MDA) and normalized to total protein. Cells were exposed to carbon black nanoparticles and Fe_2_O_3 _nanoparticles, as well as co-exposed to carbon black and Fe_2_O_3_. After 25 hours of exposure to the particles, the cells were harvested, centrifuged, and re-suspended in 100 μL of 1× phosphate buffered saline solution (pH 7.2). The suspension was then sonicated for 20 minutes to lyse the cells. After centrifugation, 100 μL of the supernatant was plated into each well of a 96-well protein binding plate. The level of MDA adduct was determined using OxiSelect MDA Adduct ELISA Kit (Cell BioLabs Inc., San Diego, CA). The appearance of MDA adduct was measured in the supernatant at 450 nm using a plate reader (Thermo electron Corp, Multiskan MCC) and compared to a standard curve containing 0–1.0 pmol/mg MDA. Lipid peroxidation was normalized to protein content.

### Statistical Analyses

Statistical analyses were performed using the SAS 9.1 software (SAS Institute Inc, Cary, NC). Each experimental value was compared to the corresponding control value for each time point. One-way ANOVA test was performed for each sampling time. When the F-test from ANOVA shows significance, the Dunnett's or Dunn's test was used to compare means from the control group and each of the groups exposed to particles. Statistical significance versus PBS control was established at levels corresponding to p-values < 0.05.

### Cellular Iron Uptake Assay

A549 cells were incubated in 7 mL of cell culture medium spiked with 3.5 μg of Fe_2_O_3 _(single exposure), In addition, cells were also incubated in 7 mL of cell culture medium spiked with 3.5 μg of Fe_2_O_3 _and 3.5 μg of carbon black (co-exposure). At 25 hours of incubation, the cell culture medium (hereafter referred to as "spent medium") was collected from the culture by pipetting. The spent medium was diluted by a factor of 100 using a calibration blank solution (5% HCl and 1% nitric acid by volume). The resultant solution/suspension was heated at 60°C for 2 hours to dissolve the residual Fe_2_O_3 _particles. After heating, the total iron content in the spent medium was measured by inductively couple plasma-atomic emission spectroscopy (ICP-AES) in a CirOs axial spectrometer (Spectro, Kleve, Germany). In addition, 7 mL of pristine cell culture medium and 7 mL of medium spiked with 3.5 μg Fe_2_O_3 _particles were also subjected to the dilution/heating procedure described above, and their iron content was measured by ICP-AES.

### Measurement of Reduction of Dissolved Fe^3+ ^Ions by Carbon Black Nanoparticles

The carbon black particle samples (38.6 ± 0.5 mg each) were weighed, and each mixed with a Fe_2_O_3 _particle sample (1.4 ± 0.2 mg). Then each mixed carbon-black/Fe_2_O_3 _pair was put in a Pyrex^® ^glass beaker. All glassware was acid washed and rinsed with deionized water before each use. To the beaker 100 mL 0.75 M sulfuric acid was added. Method blank was prepared by adding 100 mL 0.75 M sulfuric acid to an empty beaker. A carbon black reference was prepared by depositing 40 mg of carbon black in the beaker and adding 100 mL 0.75 M sulfuric to the beaker. A Fe_2_O_3 _reference was prepared by depositing 1.4 mg Fe_2_O_3 _in the beaker and adding 100 mL 0.75 M sulfuric to the beaker. The beakers containing the carbon-black/Fe_2_O_3 _pairs, the blank and the carbon-black and the Fe_2_O_3 _references were then placed in a water bath maintained at 40°C. Beakers containing the carbon-black/Fe_2_O_3 _pairs were removed from the water bath at 1, 2, 4, 8 and 16 hours. The solution in the beaker was immediately filtered using an ash-free 100-mm paper filter to remove the carbon black particles and the undissolved Fe_2_O_3 _particles. The Fe^2+ ^and Fe^3+ ^ion concentrations in the filtrate were measured using a phenanthroline-based spectrophotometric method, as described previously. [21] The method blank and the two references were taken out of the water bath at 8 hours. The solutions were passed through the filter, after which the filtrates were measured for Fe^2+ ^and Fe^3+ ^ion concentrations in the same way as for the solutions derived from the carbon-black/Fe_2_O_3 _pairs. Similar procedures had been used by other researchers to determine the Fe^3+ ^and Fe^2+ ^concentrations in aqueous solutions. [17]

## Competing interests

The authors declare that they have no competing interests.

## Authors' contributions

BG conceived the overall research idea, designed nanoparticle synthesis/characterization and the experiment on Fe^3+ ^reduction by carbon black, and wrote the paper. CS designed the cell culture, oxidative stress, and cellular iron uptake experiments and aided in writing and interpreting the results for the manuscript. RZ conducted the cell culture, oxidative stress, and metal analyses experiments. SD conducted the nanoparticles synthesis and the experiment on Fe^3+ ^reduction by carbon black.
